# Determining the incidence, risk factors and biological drivers of irritable bowel syndrome (IBS) as part of the constellation of postacute sequelae of SARS-CoV-2 infection (PASC) outcomes in the Arizona CoVHORT-GI: a longitudinal cohort study

**DOI:** 10.1136/bmjopen-2024-095093

**Published:** 2025-01-30

**Authors:** Kristen Pogreba Brown, Erika Austhof, Caitlyn M McFadden, Caroline Scranton, Xiaoxiao Sun, Ivan Vujkovic-Cviji, Dominic Rodriguez, Laura Falk, Kelly M Heslin, Gayatri Arani, Victoria Obergh, Kate Bessey, Kerry Cooper

**Affiliations:** 1 Mel and Enid Zuckerman College of Public Health; Department of Epidemiology and Biostatistics, The University of Arizona Health Sciences, Tucson, Arizona, USA; 2 School of Animal and Comparative Biomedical Sciences, The University of Arizona College of Agriculture and Life Sciences, Tucson, Arizona, USA; 3 Department of Biomedical Sciences; Research Division of Immunology, Department of Medicine, Karsh Division of Gastroenterology, Cedars-Sinai Medical Center, Los Angeles, California, USA; 4 Mel and Enid Zuckerman College of Public Health; Department of Epidemiology and Biostatistics, The University of Arizona, Arizona Health Sciences Center, Tucson, Arizona, USA

**Keywords:** Irritable Bowel Syndrome, Post-Acute COVID-19 Syndrome, COVID-19, Functional bowel disorders, EPIDEMIOLOGIC STUDIES

## Abstract

**Abstract:**

**Introduction:**

Postacute sequelae of SARS-CoV-2 infection (PASC) are extensive. Also known as long COVID, primary outcomes reported are neurologic, cardiac and respiratory in nature. However, several studies have also reported an increase in gastrointestinal (GI) symptoms and syndromes following COVID-19. This study of PASC will include extensive analyses of GI symptoms, determine if people with pre-existing irritable bowel syndrome (IBS) are at higher risk of developing PASC generally or PASC-GI, and which biomarkers are impacted and to what degree. This R01 study is being funded by the National Institute of Diabetes and Digestive and Kidney Diseases (1R01DK135483-01) from 2023 to 2028.

**Methods and analyses:**

This study combines a longitudinal epidemiologic cohort study and in-depth, novel biologic analyses. In collaboration with a pre-existing study, the Arizona CoVID-19 Cohort (CoVHORT)-GI will recruit participants based on the history of COVID infection(s), new or ongoing GI symptoms 3–6 months postinfection, and pre-existing or incident IBS diagnosis to represent five study groups for comparison and analyses. A subset (n=1000) of those recruited will submit both stool and blood samples. Both samples will undergo a novel method to quantitate humoral and mucosal immune responses to host-derived faecal communities in conjunction with magnetic bead-based separation and high-depth shotgun microbial sequencing. Stool samples will also undergo traditional microbiome analyses (diversity and abundance) and faecal calprotectin assays. Additional serum analyses will aim to determine if a proteomics-based signature exists that differentiates a unique biomarker compositional signature discriminating PASC-GI versus no PASC. All laboratory data will be linked with in-depth epidemiologic data on demographics, symptoms and chronic conditions.

**Ethics and dissemination:**

This study involves human participants and was approved by the University of Arizona Institutional Review Board (IRB (#00002332) and has been deemed minimal risk. Participants gave informed consent to participate in the study before taking part. All publications from the study will be shared back to participants along with alternative lay summaries and webinars to communicate key findings. The data management plan has been published and is publicly available online, including protocols for data requests.

**STRENGTHS AND LIMITATIONS OF THIS STUDY**

- The Arizona CoVHORT uses partnerships with public health agencies and clinics to allow for community-based recruitment and representation of non-hospitalised cases which make up the vast majority of COVID-19 cases.
- Using a pre-existing COVID-19 longitudinal cohort study for recruitment is an efficient way to collect retrospective and prospective epidemiologic data on health history, exposures and outcomes.
- Blood specimens are collected using a novel, user-friendly, at-home method and will be analysed using newly developed immunologic and proteomic methods (IgG/IgA Seq and Somalogic).
- The use of shotgun metagenomics allows for the identification of functional capability changes in the gut and not simply the absence/presence/abundance of specific microbial species.
- Most biospecimens will only be available postinfection. Ideally, samples would also be taken before and during acute infection to track individual changes and not only differences by group.

## Introduction

 Infection with SARS-CoV-2, leading to the disease COVID-19, is characterised by fever, chills, cough, shortness of breath, fatigue, and for some, gastrointestinal (GI) symptoms. SARS-CoV-2 targets ACE-2 receptors which are prolific in the GI tract^1^ intestinal inflammatory processes,^2^ gut dysbiosis,^3^ as well as neurological symptoms (eg, brain fog, sleep disturbance, stress, anxiety and depression) during their acute infection phase and beyond. These symptoms have been shown to interrupt the gut–brain axis which is often the first step in the development of disorders of gut–brain interaction (DGBI).^4^The most common DGBI is irritable bowel syndrome (IBS), with some estimates indicating that postinfectious IBS (PI-IBS) accounts for at least 10% of all IBS cases;^5 6^ while some researchers argue that a majority of IBS cases could have an infectious origin.^7^ Our prior work using data from the longitudinal Arizona CoVHORT study showed that PI-IBS occurred more frequently in persons who experienced acute GI symptoms during their COVID-19 infection compared with those without acute GI symptoms.^8^ Several studies published after our initial report have also indicated that GI symptoms during acute COVID-19 are predisposing factors for the development of PI-IBS.^9^,^12^ Another analysis from the Arizona CoVHORT found that people who reported one of several pre-existing GI conditions (IBS, chronic diarrhoea, chronic constipation, reflux, colitis, ulcerative colitis and liver disease) were 1.62 times more likely (95% CI 1.16–2.26) to develop postacute sequelae of COVID-19 (PASC)^13^ or more commonly referred to as Long COVID, which would indicate there may be an elevated risk for people with pre-existing IBS and the development of long-term sequelae following infection. Several studies have also explored faecal microbiome alterations in SARS-CoV-2-infected patients and found significant compositional differences that can remain for upwards of 6 months postinfection.^14 15^ Additionally, systematic reviews resulting in meta-analyses estimate the pooled incidence of IBS following COVID-19 is between 7.2% and 15%.^16^,^18^ However, very little is known about the epidemiology or the potential mechanism of action of SARS-CoV-2 in the gut.

Through a National Institute of Diabetes and Digestive and Kidney Diseases-funded R01 study, we propose to investigate whether there are differences in the incidence of IBS between SARS-CoV-2 cases and non-cases and discern how GI symptoms present within the broader constellation of PASC or long COVID conditions. We will also investigate whether the presence of IBS prior to SARS-CoV-2 infection results in PASC at a greater rate than for those who did not have IBS prior to infection. Finally, through biospecimen collections and analysis, we will establish the underlying mechanisms of IBS following SARS-CoV-2 infection including differences in the faecal microbiome, the host’s anticommensal immune response and protein biomarker serology. Our central hypotheses are (1) participants who were infected with SARS-CoV-2 will have a higher incidence of IBS than non-cases, (2) participants who experience GI symptoms during their acute SARS-CoV-2 infection will develop IBS at an elevated rate compared with those without acute GI symptoms and (3) post-SARS-CoV-2 patients who experience acute GI symptoms and develop PASC-IBS will have variation in the composition of the microbiome, serum proteome and anticommensal antibody profiles using a novel method (host-derived faecal communities (HFC)-IgG-seq/HFC-IgA-seq) compared with cases without IBS and healthy controls.

## Methods and analyses

### Study design and setting

Arizona CoVHORT Methods: the CoVHORT-GI study is an ancillary study of the Arizona COVID-19 (CoVHORT) longitudinal cohort study. All participants first enrol in CoVHORT, and, if eligible (described below), are provided the option to complete an additional consent form and enrol in CoVHORT-GI. CoVHORT methods have been described in detail elsewhere.^19^ Briefly, the CoVHORT was launched in May 2020 and continues to enrol participants with the goal of recruiting adult (18+ years old) SARS-CoV-2 cases and non-cases across the state. Recruitment is a multifaceted, evolving process. From June 2020 to October 2023, cases were recruited from testing centres state-wide, ensuring laboratory-confirmed cases are contacted within days of their diagnosis. This allowed us to recruit people who have tested positive or negative for SARS-CoV-2. From August 2020 to today, cases that are reported to public health agencies and complete the routine public health surveillance case investigation conducted by state and local health departments are informed about the study and sent an email invitation and consent if they opt in. Recruitment communication has also included mailed household postcards, partnerships with several other studies, traditional and social media advertisements and flyers at vaccine dispensing points across Arizona. Starting in mid-2023, recruitment efforts expanded to account for home testing and reduced centralised testing/vaccine locations. This included partnerships with local clinics for primary care and gastroenterology. All recruitment and study materials are available in English and Spanish and have received IRB approval (#2003521636).

Following initial consent into CoVHORT, participants complete surveys online through Research Electronic Data Capture (REDCap). Baseline data within the parent CoVHORT study include COVID-19 status (test and/or onset date, test type (lab or home) and result of testing), demographics, pre-existing health conditions, perceived stress, exercise, smoking, household demographics, healthcare-seeking behaviours, vaccination status (date of each dose and vaccine manufacturer) and continuing health status. Each subsequent quarterly survey contains a core set of questionnaires asking about any new SARS-CoV-2 infections and test results, new or ongoing symptoms, new diagnosis of/or changes to a chronic disease and general well-being questions. Rotating questions asked every 6–12 months include topics on grief, resilience, household infections and quality of life. All participants are asked if they have had any of 29 specific symptoms, including GI symptoms such as diarrhoea (≥3 loose/looser than normal stools/24-hour period), nausea, vomiting, abdominal pain and other (free response). For SARS-CoV-2 cases, these symptoms are in reference to the acute phase of their infection or on a symptom/quarterly survey following infection. For non-cases, they are asked to recall any of the same symptoms over the same timeframe in which cases are asked to recall symptoms.

As of July 2024, over 9000 people have completed the baseline survey and over 3700 and 2400 have completed the 12- and 24-month follow-up surveys, respectively. This study continues to recruit new participants and is currently slated to do so until at least the end of 2027 with follow-up continuing until at least 2029.

CoVHORT-GI recruitment: CoVHORT serves as a recruitment pool, but CoVHORT-GI is a separate ancillary study with an independent IRB (#00002332) and an additional associated consent form. These participants agree to have their epidemiologic data shared from CoVHORT surveys and agree to complete additional questionnaires and provide biospecimens. Participants are recruited based on their history of SARS-CoV-2 infection, GI symptoms and patient-reported IBS or other DGBI diagnosis. Recruitment began in February 2024 and will continue until March 2027.

### Participants

SARS-CoV-2 cases and non-cases: there are two primary recruitment groups for SARS-CoV-2 cases: (1) people who have tested positive in the previous 12 months and (2) participants who tested positive in the prior 4 weeks. Anyone who reports having tested positive more than 12 months prior or reports never testing positive is considered non-case for the purpose of recruitment. Because the goals of the project are to both describe the incidence of new IBS following SARS-COV-2 infection and describe any elevated risks for long COVID symptoms for people with pre-existing IBS, both groups are currently included within the eligibility criteria. Related DGBIs, such as functional dyspepsia and reflux hypersensitivity, are also eligible for recruitment. See table 1 for the inclusion criteria for each group.

**Table 1:** Description and inclusion for study groups

Group	SARS-CoV-2	GI outcome	Inclusion criteria	Sample collection timeline
1a	Case	PASC-IBS	Developed or had ongoing GI symptoms within 3–6 months of infection **AND** met the Rome IV definition of IBS no more than 18 months from infection **OR** had a provider diagnosis of IBS 6–12 months postinfection.	6–9 months postinfection (*target*)6–18 months postinfection (*eligibility window*)
1b	Case	PASC-GI	Developed persistent GI symptoms within 3–6 months of infection **AND** does not meet all necessary criteria for IBS under Rome IV.	6–9 months postinfection (*target*)6–18 months postinfection (*eligibility window*)
2	Case	None	No GI symptoms reported in previous 6 months.	6–12 months postinfection
3	Non-case	IBS	No more than 12 months from incident IBS **OR** IBS flare-up* if diagnosis more than 12 months prior	0–12 months post diagnosis **OR** 3 months post flare-up*
4	Non-case	None	No GI symptoms reported in previous 6 months.	6 months with no GI symptoms
Acute	Case (acute)	Group determined 6 months postinfection	Tested positive in last 4 weeks and agrees to provide biospecimen at two time points (acute and 6 months postinfection)	4≤weeks from positive test **AND** 6 months postinfection

* Participants with pre-existing IBS diagnosed >12 months prior who also report at least one flare-up in the past 12 months will be included in group 3.

GI, gastrointestinal; IBS, irritable bowel syndrome; PASC, postacute sequelae of SARS-CoV-2.

Patient and public involvement statement: participants were not formally involved in the development of the design of the study or research. However, this ancillary study was developed, in part, due to participant feedback following webinars and open-ended survey questions regarding long-term symptoms in the original CoVHORT study.

Consent and screening: following recruitment, potential participants are sent a consent form specific to CoVHORT-GI through REDCap. Participants are asked to consent to additional survey instruments and informed that they may be invited to provide up to two biospecimen samples (eg, blood and stool samples). The screening survey includes more detailed questions about each infection, timeline of GI symptoms, pre-existing GI conditions and management, medications prescribed (antivirals, antibiotics, GI symptom management, etc), brief quality of life assessment and willingness to provide biospecimen samples if eligible. Screening for biospecimen collections takes into account the person’s current symptoms and/or diagnoses and time from infection.

## Surveys and data collections

Once participants have enrolled, they will be asked to complete one or more additional surveys immediately following consent. On a quarterly basis, participants who report experiencing GI symptoms currently or within the past 3 months are sent the Rome IV questionnaire. The Rome IV is a standardised GI diagnostic survey tool used by gastroenterologists and other healthcare providers.^20^ Participants who report persistent abdominal symptoms or meet the criteria for IBS as determined by their responses on the ROME IV are asked to complete the IBS-Quality of Life (IBS-QoL) survey immediately following the ROME IV. The IBS-QoL was constructed and validated specifically to measure the impact of IBS associated with treatment and other factors that could have an impact on the disease course.^21^ These same participants are asked to complete the IBS Symptom Severity Scale (IBS-SSS) 3 months after the survey in which the Rome IV criteria for IBS have been met to assess changes in symptom severity over time. The IBS-SSS is a validated scoring system to better quantify the severity of a person’s symptoms by using information related to pain, distension, bowel dysfunction and quality of life (score 0–500).^22^

**Figure 1:**
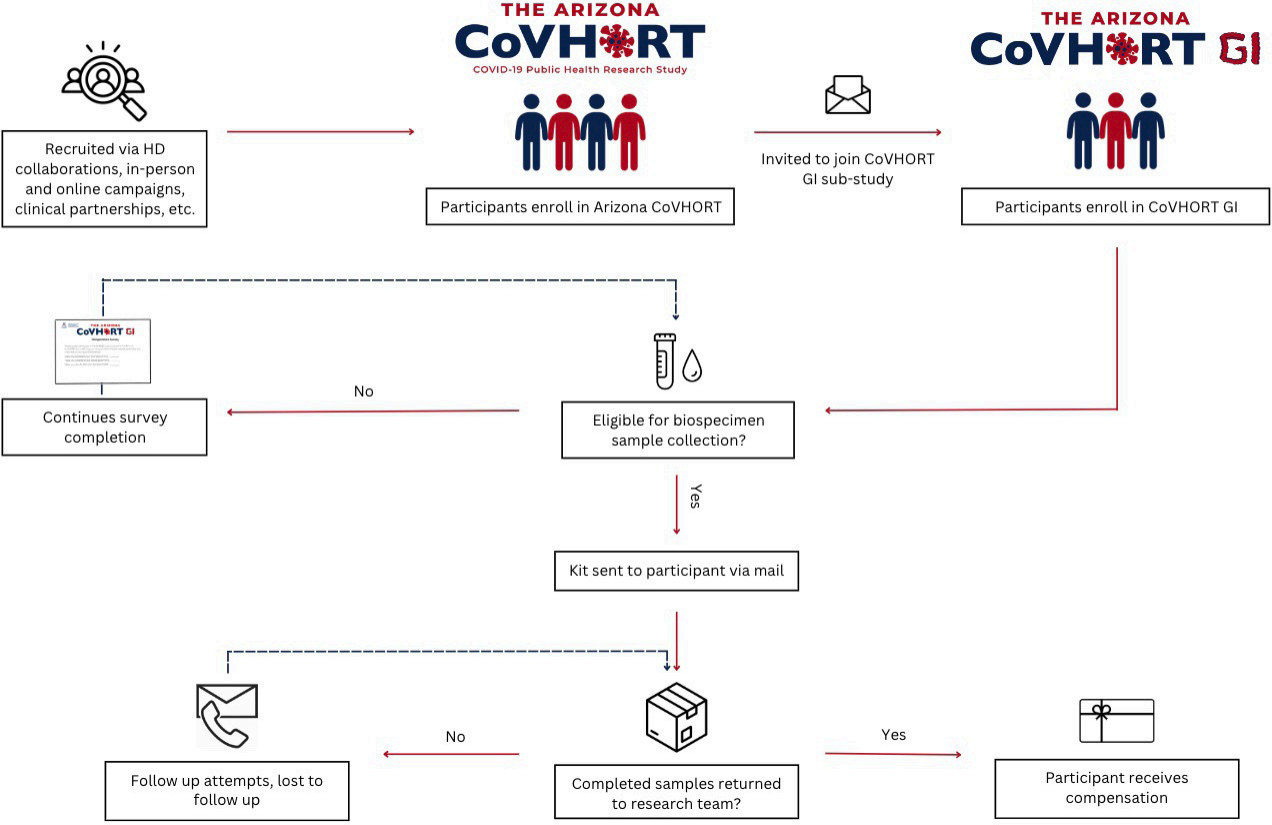
Arizona CoVHORT GI study design and recruitment process flow. Figure depicts a flowchart of how participants move from the larger study, CoVHORT, into the substudy, CoVHORT-GI. As participants complete surveys and are eligible for biospecimen collection, the flowchart shows when participants receive follow-up, their biospecimen collection kits, and finally compensation. GI, gastrointestinal; HD, health department.

Biospecimen recruitment and collection: a subset of 1000 participants will be recruited to provide stool and blood biospecimens based on the criteria provided in table 1. Participants will be sent a kit to allow for at-home collection of all samples and when the kit is returned, they will receive a USD $50 Amazon gift card via email (figure 1). Detailed biospecimen logistics are provided in the online supplemental material appendices A-C.

### Variables

GI and associated DGBI symptoms: GI symptoms included in survey questions include diarrhoea (>3 loose stools in a 24-hour period), bloating, constipation, blood in stool, blood on toilet paper, feeling of not emptying bowels, waking up in the night from urgency, difficulty swallowing, feeling full quickly while eating, acid reflux or heartburn, abdominal pain and anorexia or weight loss (>10 pounds). Associated psychological factors include anxiety or depression. Participants are considered GI symptom-positive if they report one or more GI symptoms, lasting three months or longer. This time period was selected to be consistent with both the Rome IV criteria for IBS diagnoses and the new recommended definition of long COVID that includes ongoing, intermittent or continuous symptoms of 3 months or more postinfection.^23^

DGBI, including IBS: patients who report recurrent abdominal pain one or more times a week (lasting 3 months or more), related to at least two of the following (1) defecation, (2) change in frequency of stool or (3) change in form/appearance of stool lasting at least 6 months on the Rome IV meet the standard definition for IBS. Participants who report any recurrent abdominal pain associated with at least one of the following (1) defecation, (2) change in frequency of stool or (3) change in form/appearance of stool lasting at least 6 months are defined as ‘Persistent abdominal symptoms not meeting the IBS definition’ or PASC-GI for this study.

### Biospecimen laboratory analyses

**Figure 2:**
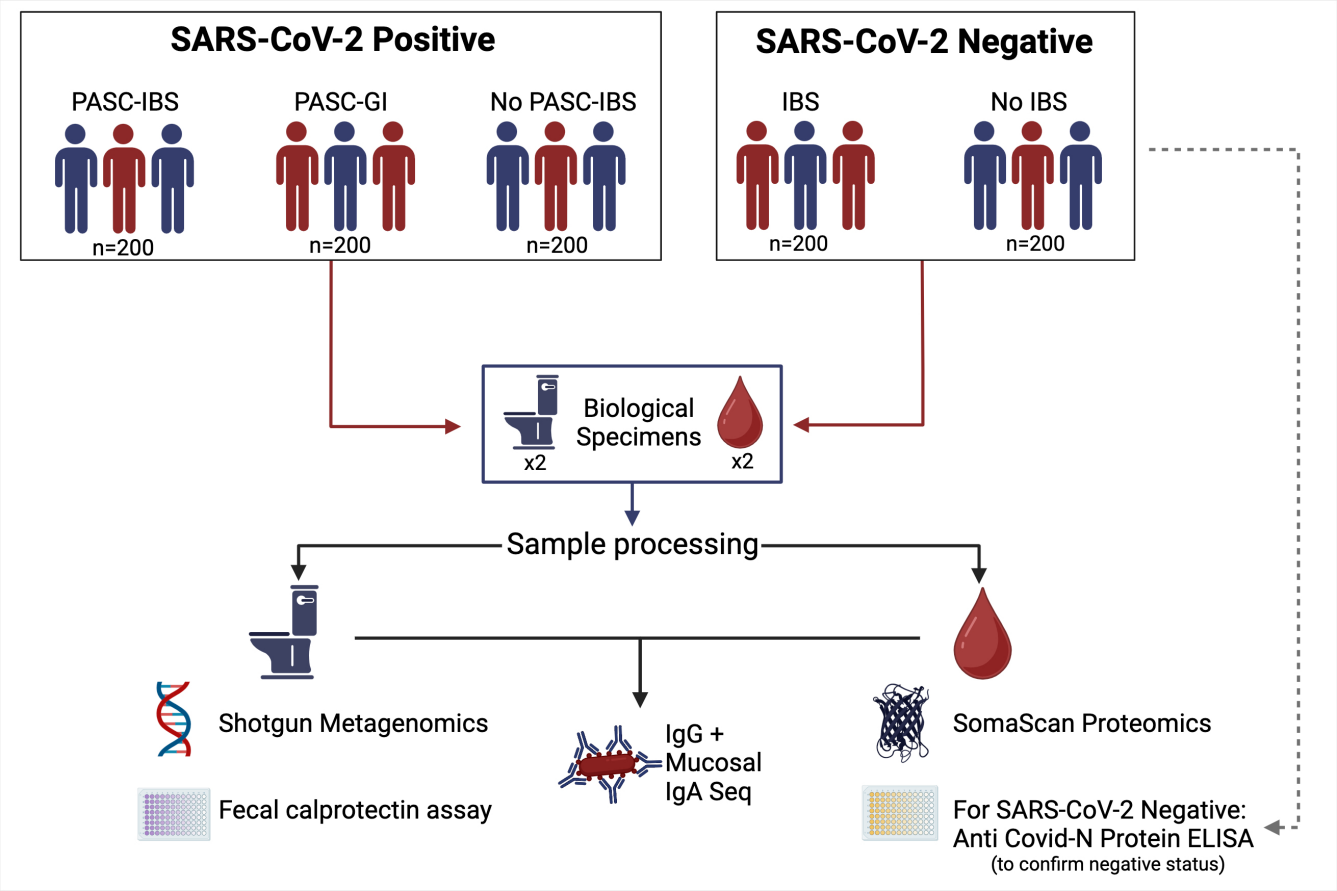
Figure depicts a flowchart of the 1000 eligible participants who will complete biospecimen collections, which subgroup they are assigned to and which sample processing steps are completed. Samples include stool and blood specimens which are then tested for shotgun metagenomics, faecal calprotectin assays, antibodies, SomaScan proteomics and a protein ELISA to confirm COVID-negative status. GI, gastrointestinal; IBS, irritable bowel syndrome; PASC, post-acute sequelae of COVID-19.

Blood specimens will be self-collected using the Tasso+ devices, where each device allows for 500 µL–600 µL of blood collection without the need for anticoagulants and immediate serum separation. Serum from sample #1 will be used for antimicrobial antibody profiles (HFC-IgG-seq/HFC-IgA-seq) of the participants to their gut microbiome, while sample #2 will be reserved for a high-dimensional serum proteomic array of >10 000 proteins (SomaScan Assay; SomoLogic). One of the two stool samples will be collected in DNA/RNA Shield (Zymo) to protect against DNA degradation during transport and then will be used for shotgun metagenomics including DNA extraction, sequencing and computational analysis to characterise the composition and functional capabilities of the faecal microbiome. A second stool culture will be collected with no preservative and will be processed for the HFC-IgG-seq/HFC-IgA-seq to assess the participant’s immune response against their own faecal microbiome (figure 2).

DNA extraction: in a biosafety cabinet, approximately 250 mL of the faecal sample from the participant’s received DNA/RNA Shield Faecal Collection tube will be transferred to the PowerBead Pro Tube. DNA will be extracted from the stool sample using the PowerSoil Pro Kit (Qiagen) per the manufacturer’s instructions with the following modifications: (1) after 800 mL of solution CD1 is added, the samples will be incubated at 65°C for 10 min; (2) samples will be centrifuged for 15 s following the addition of solution CD3; and (3) during the final step, 50 mL of solution C6 will be added to the spin column and centrifuged twice for a total of approximately 100 mL eluted DNA. The DNA from each participant’s sample will be quantified using a Qubit Fluorometer, and the appropriate amount of input DNA will be used to prepare sequencing libraries as described below.

Sequencing library preparation: sequencing libraries will be prepared for both Illumina (short-read) and Oxford Nanopore (long-read) sequencing technologies, which will allow for hybrid assemblies, thus improving the completion of microbial genomes from the samples. Illumina barcoded sequencing libraries will be prepared using the DNA Prep Kit (Illumina) per the manufacturer’s instructions with the following modification: (1) the number of PCR cycles will be determined by the input DNA concentrations as follows: 6.25–24.9 ng/mL=6 cycles, 2.5–6.24 ng/mL=8 cycles and 0.25–2.49 ng/mL=12 cycles. Input DNA concentrations of 25–125 ng/mL will be targeted. Oxford Nanopore barcoded sequencing library will be generated using the Ligation Sequencing kit V14 (Oxford Nanopore) per the manufacturer’s instructions. Libraries for a particular technology will be pooled in equal molar ratios, and pooled barcoded Illumina libraries will be sequenced on an Illumina NovaSeq S4 sequencer, while pooled barcoded Oxford Nanopore libraries will be sequenced on an Oxford Nanopore PromethION P2 solo device. Each of the stool samples will get at least 2 Gb of sequencing data per sequencing technology for a total of 4 Gb per sample. Initially, the sequence data will be quality checked and filtered using fastp software^24 25^ to generate high-quality filtered sequence reads that will be used for an initial taxonomic profiling of the different samples. Particularly, fastp detects and removes the polyG tails that are often associated with Illumina NextSeq/NovaSeq data, while also doing the quality filtering of the reads. Due to variations in the quality of the sequence technologies, different quality filters will be applied, as the Illumina sequence reads will be filtered at the QXX score, whereas the Oxford Nanopore reads will be filtered at the QXX score. Shotgun metagenomic data analysis will be conducted as described above.

IgG/IgA Seq: we are using a novel assay that can quantitate humoral and mucosal immune responses to HFC in conjunction with magnetic bead-based separation and high-depth shotgun microbial sequencing. HFC-IgG-seq/HFG-IgA-seq will be conducted on serum and stool samples from patients following the methods outlined by Vujkovic-Cvijin *et al*^26^ ^55^ with slight modifications: (1) the patient’s serum (IgG-seq+IgA seq) and stool (IgA-seq) samples will be used directly against their faecal sample (faecal microbiome) for immune response to commensal microbes; (2) shotgun sequencing will be used instead of 16S rRNA amplicon sequencing expanding the potential to identify other microbial commensals (eg, fungal, protozoan, etc.) that the host raises an immune response to during PASC-IBS. Briefly, collected patient serum samples will have complement inactivated by heat treatment at 56°C for 30 min and then diluted 1:50 prior to incubation with processed stool samples. Stool samples will be processed by resuspending 200 mg in 1% bovine serum albumin in phosphate-buffered saline (w/v), centrifuged at 200 x g for 1 min to remove large particles and then separated into four aliquots: one aliquot for HFC-IgG-seq (incubate with serum at 4°C for 30 min), one aliquot for HFC-IgA-seq (incubate with serum at 4°C for 30 min), one aliquot for mucosal HFC-IgA-seq (processed stool samples with no serum incubation) and one aliquot for pre-enrichment shotgun metagenomics (as described above in the shotgun metagenomics section). The sample will be stained with anti-IgG Phycoerythrin (PE) (Miltenyi, 1:50 dilution) or anti-IgA PE (Miltenyi, 1:50 dilution) antibodies, as well as with the bacterial cell stain SYTO-62 (Invitrogen, 1:500 dilution) and then used for magnetic column separation using anti-PE microbeads (Miltenyi, 1:20 dilution). This will be repeated at least twice to increase IgG/IgA enrichment and then processed for shotgun metagenomic sequencing as described previously. An aliquot of the enriched samples will be used for isotyping of the antibodies and will be assessed using flow cytometry (figure 2).

Serum proteomics: the primary analysis will be to define a proteomics-based signature that differentiates a unique biomarker compositional signature that discriminates PASC-IBS versus no PASC. Host serum biomarkers will be assessed using high-dimensional serum proteomic arrays using the SomaScan Assay v4.1 (SomaLogic, Boulder, CO). The SomaScan Assay is an aptamer-based protein-capture system that measures approximately 10 000 unique human protein analytes, including major domains of both cellular and humoral immunity as well as GI domain proteins. Our group has used a previous version of this technology in the IBD discovery effort.^27^ In short, 55 uL of the patient’s serum sample collected with the Tasso+ devices will be shipped to Somalogic (Boulder, Colorado) for the SomaScan Assay. Proteomic data from each participant will be returned and used for analysis as described previously (figure 2).

Additional assays: participant faecal samples that are collected without any preservative will also be used to assess the intestinal inflammation by quantifying the calprotectin levels in the sample. Faecal calprotectin levels will be determined using the LLC Immunchrom Calprotectin ELISA for Stool Kit (ILEX Life Sciences) per the manufacturer’s instructions. Additionally, participants in the COVID-19 non-case group will be confirmed by screening blood samples for the presence of antibodies to the SARS-CoV-2 N protein, which are only generated through natural infection and not immunisation. N protein antibody levels will be quantified using the Anti-SARS-CoV-2 N protein Human IgG ELISA Kit (ProteinTech) per the manufacturer’s instructions.^28^

### Statistical methods

Data collected through CoVHORT-GI surveys and biospecimen analyses will be merged with participant data collected through CoVHORT (eg, data on acute symptoms for an infection, vaccination date(s), history of pre-existing conditions and demographics). We will summarise participant data including exposures (SARS-CoV-2), outcomes (IBS, GI symptoms), potential risk factors (acute symptoms, pre-existing conditions, vaccinations, etc) and possible confounders (gender, age, ethnicity). We will use logistic regression to estimate the incidence of IBS following a SARS-CoV-2 infection reporting unadjusted and adjusted ORs and 95% CIs. PASC-IBS-specific incidence rates for the case population, as well as rates stratified by demographic characteristics (eg, ethnicity, age and gender), will be calculated. We will estimate the association of possible risk factors using logistic regression models adjusting for demographic, clinical or exposure variables. Because initial symptom onset will be known, time to PASC-IBS will be modelled using survival methods, including Kaplan-Meier curves and Cox regression models. We will use Cox regression to model the time to resolution of PASC-IBS and to estimate associations with potential risk factors in individuals with PASC-IBS, if the sample size permits. Exploratory regression models will use other patient-reported outcomes to investigate the effects of PASC-IBS on lifestyle, physical manifestations and functional disability.

To determine the role of pre-existing IBS, we will use data from participants who have indicated they were diagnosed with IBS prior to SARS-CoV-2 infection to determine the rate of PASC (examining PASC as it relates to the incidence of new non-GI symptoms as well as reports of worsening of existing GI symptoms) compared with the incidence in those without pre-existing IBS. In addition to the development of PASC, we can examine broader questions such as medications or other treatments used and specific changes in GI symptoms. Our central hypothesis is that participants with pre-existing IBS have an increased risk of developing PASC (even for non-GI symptoms) compared with people without pre-existing IBS. We will use the Cox proportional hazards model (univariate) to determine if IBS increases the risk of developing PASC, and hazard ratios (95% CIs) will be examined, accounting for differences between groups and assessing relevant confounders and effect modifiers. In prior studies using data from the CoVHORT, we have found that pre-existing chronic conditions (presence and specific types) are effect modifiers of the association between COVID-19 and PASC. We will explore stratified or interaction analyses for chronic condition status as appropriate.

Biospecimen data analyses: the outcomes of the biospecimen data analyses will be to (1) characterise faecal microbiome compositional and functional capability differences that are unique to participants who develop PASC-IBS or PASC-GI; (2) categorise anticommensal immune reactions unique to people who develop PASC-IBS or experience long-term GI issues following SARS-CoV-2 infection and (3) identify serum biomarkers that are unique to those participants who develop PASC-IBS or experience long-term GI symptoms following SARS-CoV-2 infection.

Data analyses for host microbiome: sequencing data for each sample will be initially used for a general taxonomic profiling of the samples by running the filtered reads through taxonomic classification system, Kraken2 software,^29 30^ using a custom database that includes the entire bacterial, fungal, viral, archaeal, protozoan and non-redundant (nr) nucleotide databases from the National Centre for Biotechnology Information using an onsite high-performance computing system. Kraken2 taxonomic classifications for each sample will be exported using Kraken-biom^31^ into R, and initial analysis of the taxonomic profile, statistical analysis and visualisation will be performed using the phyloseq,^32 33^ microbiome,^34^ microbiome utilities^35 36^ and vegan^37^ packages. Additional, taxonomic classification will be double-checked using Centrifuge,^38^ Kaiju^39^ and MetaMaps.^40^ Next, the high-quality filtered^41^ sequence reads for each sample will be run through the most up-to-date bioBakery pipeline^41^ for each sequencing technology and also as a hybrid through the Metagenomics workflow for hybrid assembly, differential coverage binning metatranscriptomics and pathway analysis (MUFFIN) pipeline.^42^ The bioBakery and MUFFIN pipelines will confirm the taxonomic profile of the samples and provide functional profiling of the different microbiome samples. Results for the initial Kraken2, Centrifuge, Kaiju, MetaMaps analysis, bioBakery and MUFFIN pipelines will be compared for an overall confirmation of the composition of the gut microbiome. Variation in compositional and abundance data between different participant groups for MUFFIN, bioBakery, Kraken2, Centrifuge, Kaiju and MetaMaps analysis will be determined using the Analysis of Compositions of Microbiomes with Bias Correction (ANCOMBC) package in R.^43^

The use of shotgun metagenomics will allow for a detailed examination of the functional capabilities (ie, presence or absence of specific genes) of the microbial communities (eg, beyond just identification of particular bacteria like 16S rRNA amplicon sequencing) between the different groups. Additionally, standalone hybrid assemblies will be done using two different assemblers: (1) OPERA-MS^44^ and (2) hybridSPAdes^45^ for each sample to compare against the assemblies of the above-mentioned pipelines. Each of the assemblies and bins generated from either standalone software or pipelines will be quality-checked using both metagenomic Quality Assessment Tool for Genome Assemblies (metaQUAST)^46^ and CheckM^47^ either independently or as part of the pipeline. All contigs generated from either pipeline or the standalone assemblies will be screened against various databases and other tools for further assessing the functional capabilities of the gut microbial communities including but not limited to Ghost KEGG Orthology and Links Annotation (GhostKOALA; Kyoto Encyclopedia of Genes and Genomes (KEGG) mapping),^48^ Carbohydrate-Active enZymes Database (CAZy; carbohydrate-active enzymes)^49^ and Metabolite Observations and Species Abundances version 2 (MIMOSA2; metabolite production).^50^ The composition and functional capabilities (carbohydrate utilisation, metabolite production, etc) of the participant’s faecal microbiome will be determined for each participant and compared across the five comparison groups in order to determine if this data can discriminate PASC-IBS and/or PASC-GI case status.

Variation in compositional and abundance data between the groups will be determined using the ANCOMBC package in R.^43^ This data will also be compared against the HFC-IgG-seq/HFC-IgA-seq (see below) results to determine potential faecal microbial functional capabilities that are altered between groups due to a raised immune response, particularly in the PASC-IBS group.

Data analyses for serum biomarkers: for the SomaScan analysis, we will employ a range of analysis methods to evaluate differential protein-set abundancies between IBS patients and healthy controls. We will aim for a discovery approach that may be later validated with expanded cohorts or studies. SARS-CoV-2 exposure and PASC-IBS case status will be used as previously defined. To achieve discriminatory biomarker compositions between the relevant comparator groups, we will use supervised methods and randomly split the full data into training (80%) and testing (20%) sets to verify the prediction performance for IBS status using the identified protein biomarkers. The area under the receiver operating characteristic curves will be used as the evaluation metric of prediction performance. SARS-CoV-2 exposure and PASC-IBS cases status will be used as previously defined. SomaScan data will undergo quality control and transformation based on bioinformatics standards. Specifically, data is checked for batch effects with respect to experimental and demographic parameters using principal component analysis. Data will be log2‐transformed within each sample. Differential protein expression analyses will be performed using the R‐based *limma* software package. The data will be adjusted for age and gender as well as plate differences during sample analysis. The results will be used to identify proteins significantly associated with IBS-case status using a Benjamini‐Hochberg false discovery rate threshold of 1% and an absolute per cent change of ≥25% between patients with PASC-IBS and IBS (-). Canonical pathways from the Molecular Signatures Database (MSigDB) will be considered. Based on their predictive performance in a 5-fold cross-validation technique performed on the training data, Slow Off-Rate Modified Aptamers (SOMAmers) will be ordered from most to least predictive of IBS status, and pathways enriched at the top of the list will be identified by using the Kolmogorov-Smirnov statistics.

Clinical and policy implications: the proposed work is a large, population-based longitudinal cohort study conducted within a diverse population and is the optimal strategy for comparing healthy, uninfected individuals and those with mild or severe COVID-19. State-wide data will allow for greater generalisability than clinic-based data collection studies or case-control designs. Our approach facilitates rapid data collection and analysis to ascertain any differences in outcomes by risk factors. A majority of studies have focused on hospitalised patients, which represent the minority of people who have been infected with SARS-CoV-2. The Arizona CoVHORT/CoVHORT-GI represents the non-hospitalised case majority.

As described, we will investigate sex as a variable to ascertain the reasons that men may have a higher risk for COVID-19 at each phase of the disease: incidence, progression and mortality while women have been reported to have a higher incidence of PASC. Furthermore, there is a known association between IBS and female sex which will be important to consider in PASC-IBS. This variable considers whether there is a biological, innate factor that drives the progression to PASC differentially between men and women, or whether there is an environmental exposure that may explain some of the differences observed to date. We will conduct stratified analyses of exposures and outcomes by sex to obtain granular information on the variables that have the strongest association for men and women separately.

One of the biggest challenges with studying PASC/long COVID is the current lack of a reliable biomarker for diagnosis or characterising elevated risk. At the completion of this study, we hope to have a better understanding of the potential differences that are unique to PASC-IBS and/or PASC-GI for the blood protein analytes (proteomics), composition and functional capabilities of the faecal microbiome and identification of humoral and mucosal immune responses to members of the faecal microbiome. Thus, providing a strong foundation of the potential mechanisms associated with PASC-IBS that could result in the development of potential treatments and/or prophylaxis for high-risk patients. Additionally, the study also provides an opportunity to further explore the role of the gut microbiome beyond just PASC-IBS and IBS in general, but other symptoms of PASC as well. It has been hypothesised that numerous symptoms associated with PASC can be linked back to the gut microbiome,^51^ so collection of this extensive epidemiological data along with these biospecimens will allow for the expansion of the analysis to explore the gut microbiome in other aspects of PASC/long COVID.

## Ethics and dissemination

This study has been reviewed and approved by the University of Arizona Human Subjects IRB and found to be minimal-risk human subjects research (#00002332). The parent study, the Arizona CoVHORT, underwent IRB review in 2020 and was deemed minimal risk and exempt (no additional review needed for survey changes and recruitment materials) (2003521636). Dr Pogreba-Brown is the PI of both studies, and all personnel are listed on both IRBs to ensure all data shared is protected under both protocols. Data are collected and managed through REDCap (Vanderbilt University, Nashville, Tennessee), a Health Insurance Portability and Accountability Act (HIPAA)-approved Health Information Technology for Economic and Clinical Health (HiTECH) compliant system. For each follow-up questionnaire, a unique survey invitation link is emailed through REDCap to study participants. Patient and Public Involvement: Results from this study will be made publicly available through peer-reviewed journal articles and scientific conferences on a continuous basis as data allows. All publications will follow the Strengthening the Reporting of Observational Studies in Epidemiology checklist for cohort studies.^52^ The data management plan for this study, including data availability options, is available online through Data Management Plan Tool (DMPTool).^53^ We will also communicate study findings with our participants through semiannual newsletters that provide a lay summary and infographics (where appropriate) for all papers and open-access links to the full publication.

## Supplementary material

10.1136/bmjopen-2024-095093 online supplemental file 1

10.1136/bmjopen-2024-095093 online supplemental file 2

10.1136/bmjopen-2024-095093 online supplemental file 3

## References

[1] Beyerstedt S, Casaro EB, Rangel ÉB (2021). COVID-19: angiotensin-converting enzyme 2 (ACE2) expression and tissue susceptibility to SARS-CoV-2 infection. Eur J Clin Microbiol Infect Dis.

[2] Effenberger M, Grabherr F, Mayr L (2020). Faecal calprotectin indicates intestinal inflammation in COVID-19. Gut.

[3] Zuo T, Zhang F, Lui GCY (2020). Alterations in Gut Microbiota of Patients With COVID-19 During Time of Hospitalization. Gastroenterology.

[4] Mukhtar K, Nawaz H, Abid S (2019). Functional gastrointestinal disorders and gut-brain axis: What does the future hold?. World J Gastroenterol.

[5] Halvorson HA, Schlett CD, Riddle MS (2006). Postinfectious irritable bowel syndrome--a meta-analysis. Am J Gastroenterol.

[6] Thabane M, Kottachchi DT, Marshall JK (2007). Systematic review and meta-analysis: The incidence and prognosis of post-infectious irritable bowel syndrome. Aliment Pharmacol Ther.

[7] Klem F, Wadhwa A, Prokop LJ (2017). Prevalence, Risk Factors, and Outcomes of Irritable Bowel Syndrome After Infectious Enteritis: A Systematic Review and Meta-analysis. Gastroenterology.

[8] Austhof E, Bell ML, Riddle MS (2022). Persisting gastrointestinal symptoms and post-infectious irritable bowel syndrome following SARS-CoV-2 infection: results from the Arizona CoVHORT. Epidemiol Infect.

[9] Ghoshal UC, Ghoshal U (2023). Gastrointestinal involvement in post-acute Coronavirus disease (COVID)-19 syndrome. Curr Opin Infect Dis.

[10] Yamamoto R, Yamamoto A, Masaoka T (2023). Early symptoms preceding post-infectious irritable bowel syndrome following COVID-19: a retrospective observational study incorporating daily gastrointestinal symptoms. BMC Gastroenterol.

[11] Marasco G, Cremon C, Barbaro MR (2022). Prevalence of Gastrointestinal Symptoms in Severe Acute Respiratory Syndrome Coronavirus 2 Infection: Results of the Prospective Controlled Multinational GI-COVID-19 Study. Am J Gastroenterol.

[12] Wang Y-N, Zhou L-Y, Huang Y-H (2024). The incidence and predisposing factors for irritable bowel syndrome following COVID-19: a systematic review and meta-analysis. Eur J Gastroenterol Hepatol.

[13] Jacobs ET, Catalfamo CJ, Colombo PM (2023). Pre-existing conditions associated with post-acute sequelae of COVID-19. J Autoimmun.

[14] Liu Q, Mak JWY, Su Q (2022). Gut microbiota dynamics in a prospective cohort of patients with post-acute COVID-19 syndrome. Gut.

[15] Zhang D, Zhou Y, Ma Y (2023). Gut Microbiota Dysbiosis Correlates With Long COVID-19 at One-Year After Discharge. J Korean Med Sci.

[16] Mathur A, Shams U, Mishra P (2024). Post-infection irritable bowel syndrome following Coronavirus disease-19: A systematic review and meta-analysis. Indian J Gastroenterol.

[17] Marasco G, Maida M, Cremon C (2023). Meta-analysis: Post-COVID-19 functional dyspepsia and irritable bowel syndrome. Aliment Pharmacol Ther.

[18] Wang Z, Peng Y, Chen M (2023). The Prevalence of Irritable Bowel Syndrome after Severe Acute Respiratory Syndrome Coronavirus 2 Infection and Their Association: A Systematic Review and Meta-Analysis of Observational Studies. JCM.

[19] Catalfamo CJ, Heslin KM, Shilen A (2021). Design of the Arizona CoVHORT: A Population-Based COVID-19 Cohort. Front Public Health.

[20] Rome Foundation Rome iv criteria. https://theromefoundation.org/rome-iv/rome-iv-criteria.

[21] Drossman DA, Patrick DL, Whitehead WE (2000). Further validation of the IBS-QOL: a disease-specific quality-of-life questionnaire. Am J Gastroenterol.

[22] Francis CY, Morris J, Whorwell PJ (1997). The irritable bowel severity scoring system: a simple method of monitoring irritable bowel syndrome and its progress. Aliment Pharmacol Ther.

[23] Committee on Examining the Working Definition for Long COVID, Board on Health Sciences Policy, Board on Global Health (2024). A long COVID definition: a chronic, systemic disease state with profound consequences.

[24] Chen S, Zhou Y, Chen Y (2018). fastp: an ultra-fast all-in-one FASTQ preprocessor. Bioinformatics.

[25] Chen S (2023). Ultrafast one-pass FASTQ data preprocessing, quality control, and deduplication using fastp. Imeta.

[26] Vujkovic-Cvijin I, Welles H, Ha CWY Systemic igg repertoire as a biomarker for translocating gut microbiota members. Immunology.

[27] Torres J, Petralia F, Sato T (2020). Serum Biomarkers Identify Patients Who Will Develop Inflammatory Bowel Diseases Up to 5 Years Before Diagnosis. Gastroenterology.

[28] Swartz MD, DeSantis SM, Yaseen A (2023). Antibody Duration After Infection From SARS-CoV-2 in the Texas Coronavirus Antibody Response Survey. J Infect Dis.

[29] Wood DE, Lu J, Langmead B (2019). Improved metagenomic analysis with Kraken 2. Genome Biol.

[30] Lu J, Rincon N, Wood DE (2022). Metagenome analysis using the Kraken software suite. Nat Protoc.

[31] Dabdoub SM (2016). kraken-biom: Enabling interoperative format conversion for Kraken results (Version 1.2). https://github.com/smdabdoub/kraken-biom.

[32] McMurdie PJ, Holmes S (2015). Shiny-phyloseq: Web application for interactive microbiome analysis with provenance tracking. Bioinformatics.

[33] McMurdie PJ, Holmes S (2013). phyloseq: an R package for reproducible interactive analysis and graphics of microbiome census data. PLoS One.

[34] Lahti L, Shetty S (2017). (Bioconductor, 2017). tools for microbiome analysis in r. microbiome package version 1.23.1. http://microbiome.github.com/microbiome.

[35] Lahti L, Shetty S (2017). Tools for microbiome analysis in R. version 2.1.28.

[36] Shetty S, Lahti L (2022). Microbiomeutilities: microbiomeutilities: utilities for microbiome analytics. R package version 1.00.17.

[37] Oksanen FJ, Simpson GL, Blanchet FG (2017). Vegan: community ecology package.

[38] Kim D, Song L, Breitwieser FP (2016). Centrifuge: rapid and sensitive classification of metagenomic sequences. Genome Res.

[39] Menzel P, Ng KL, Krogh A (2016). Fast and sensitive taxonomic classification for metagenomics with Kaiju. Nat Commun.

[40] Dilthey AT, Jain C, Koren S (2019). Strain-level metagenomic assignment and compositional estimation for long reads with MetaMaps. Nat Commun.

[41] Beghini F, McIver LJ, Blanco-Míguez A (2021). Integrating taxonomic, functional, and strain-level profiling of diverse microbial communities with bioBakery 3. Elife.

[42] Van Damme R, Hölzer M, Viehweger A (2021). Metagenomics workflow for hybrid assembly, differential coverage binning, metatranscriptomics and pathway analysis (MUFFIN). PLoS Comput Biol.

[43] Lin H, Peddada SD (2020). Analysis of compositions of microbiomes with bias correction. Nat Commun.

[44] Bertrand D, Shaw J, Kalathiyappan M (2019). Hybridmetagenomic assembly enables high-resolution analysis of resistancedeterminants and mobile elements in human microbiomes.Nat Biotechnol.

[45] Antipov D, Korobeynikov A, McLean JS (2016). hybridSPAdes: an algorithm for hybrid assembly of short and long reads. Bioinformatics.

[46] Mikheenko A, Saveliev V, Gurevich A (2016). MetaQUAST: evaluation of metagenome assemblies. Bioinformatics.

[47] Parks DH, Imelfort M, Skennerton CT (2015). CheckM: assessing the quality of microbial genomes recovered from isolates, single cells, and metagenomes. Genome Res.

[48] Kanehisa M, Sato Y, Morishima K (2016). BlastKOALA and GhostKOALA: KEGG Tools for Functional Characterization of Genome and Metagenome Sequences. J Mol Biol.

[49] Drula E, Garron M-L, Dogan S (2022). The carbohydrate-active enzyme database: functions and literature. Nucleic Acids Res.

[50] Noecker C, Eng A, Muller E (2022). MIMOSA2: a metabolic network-based tool for inferring mechanism-supported relationships in microbiome-metabolome data. Bioinformatics.

[51] Su Q, Lau RI, Liu Q (2024). The gut microbiome associates with phenotypic manifestations of post-acute COVID-19 syndrome. Cell Host Microbe.

[52] Elm E von, Altman DG, Egger M (2007). Strengthening the reporting of observational studies in epidemiology (STROBE) statement: guidelines for reporting observational studies. BMJ.

[53] Austhof E (2024). GI-pasc in the arizona covhort [internet]. Dmptool. https://dmphub.uc3prd.cdlib.net/dmps/10.48321/D1B2B0DEE9.

