# Supplementary material for: Determining the incidence, risk factors and biological drivers of irritable bowel syndrome (IBS) as part of the constellation of postacute sequelae of SARS-CoV-2 infection (PASC) outcomes in the Arizona CoVHORT-GI: a longitudinal cohort study
Source: BMJ Open. 2025 Jan 30;15(1):e095093. doi: 10.1136/bmjopen-2024-095093 (PMC11784208; doi:10.1136/bmjopen-2024-095093)
Supplement: online supplemental file 1 [file bmjopen-15-1-s001.docx]

# **SUPPLEMENTARY FILES**

# **Appendix A – Detailed Biospecimen Shipping and Receiving**

Participants will be contacted via phone and/or email to 1) confirm their address for shipping biospecimen kit; and 2) briefly outline the biospecimen collection process. Once completed, a participant-linked barcode will be generated and each component of the kit is tagged. This also allows the Cooper laboratory (microbiology research laboratory conducting the various assays) to be blinded to participants' information and case status during the collection, processing, and analysis of all stool and blood samples. Participants will be shipped a pre-labeled FedEx box with ice packs and specimen collection instructions in both English and Spanish for both stool and blood collection (Appendices B and C). Each package will also include:

1. Two tubes for stool specimens: (1) a basic stool culture collection tube without preservative for conducting the host-derived fecal communities IgG-seq (HFC-IgG-seq) and IgA-seq (HFC-IgA-seq) analysis (described below; Fisher Scientific); and (2) a DNA/RNA Shield Fecal Collection tube for DNA extraction of the fecal sample for conducting the shotgun metagenomics (Zymo Research).
2. Two Tasso+ kits, each containing a Tasso+ device, IVD collection tube, gel pack warmer, alcohol swabs, and bandage, as well as additional blood collection instructions (Tasso, Inc) to collect two separate ~500uL blood samples. One sample will be utilized for HFC-IgG-seq analysis and the other blood sample for biomarker/proteomic analysis (described below).
3. A Commode Specimen Collector (toilet hat; Fisher Scientific) to facilitate stool collection for both stool specimens.

A biospecimen collection form will be included in the kit for participants to complete will be used to indicate date(s) of sample collection and return shipment. Participants will be notified via email once their kit has been tendered to FedEx and requested to return their completed kit or notify the research team within three weeks of delivery that their biospecimen collection is delayed and when specimens can be expected. Up to three follow-up notifications offering additional sample collection support will be sent if the research team has not received a completed kit or notification of delayed sample collection within this time frame. Once received by the Cooper laboratory, completed samples and associated collection dates identified via generic kit barcodes will be logged in a secure shared file via the barcode scanning system. Returned samples will then be checked into the study database by additional research personnel, linking kit IDs to existing participant records. Participant compensation for completed biospecimen sample collections will be sent via email.
