# Supplementary material for: Determining the incidence, risk factors and biological drivers of irritable bowel syndrome (IBS) as part of the constellation of postacute sequelae of SARS-CoV-2 infection (PASC) outcomes in the Arizona CoVHORT-GI: a longitudinal cohort study
Source: BMJ Open. 2025 Jan 30;15(1):e095093. doi: 10.1136/bmjopen-2024-095093 (PMC11784208; doi:10.1136/bmjopen-2024-095093)
Supplement: online supplemental file 2 [file bmjopen-15-1-s002.pdf]

THE ARIZONA

CoVHORT GI

SAMPLE COLLECTION KIT INSTRUCTIONS

If any of these items are missing from your kit, please call us at (520) 626-1678 or email us at CoVHORT-GI@arizona.edu

1 Part One: Inventory & Preparation

Content Checklist

Ensure your kit contains the following items:

- (1) Fecal collection tube
- (1) DNA/RNA Shield fecal collection tube
- (2) Tasso+ blood sample collection kits
- (1) Pair of disposable gloves
- (1) Commode (toilet hat)
- (1) Ice pack
- (1) Specimen sealable bag
- (1) Insulated bubble mailer
- (1) FedEx pak (poly mailer)
- (1) Biospecimen survey

General Preparation

- Read all instructions **before** collecting samples
- Place ice pack in freezer for a minimum of 4 hours or until frozen
- Plan to collect stool samples **before** collecting blood samples
- Store all samples in refrigerator before shipping - **DO NOT** store samples in the freezer!
- Plan to return kit within 24 hours after collecting blood samples
- Try to be well-hydrated before collecting blood samples
- Collect and ship samples early in the week if possible

2 Part Two: Stool Sample Collection

Prepare stool container

1. Wash hands and don disposable gloves
2. Place commode (toilet hat) onto rim of toilet seat, then close toilet seat to hold the hat in place
   a. *If you need to pee, do this **before** placing commode on toilet*
3. Sit on the toilet to have a bowel movement (poop)

Do not let the sample go directly into the toilet bowl & do not pee on your stool sample!

Collect Stool Sample

Stool Sample Collection #1 (DNA/RNA Shield - brown cap)	Stool Sample Collection #2 (Fecal Collection Tube - blue cap)
4. Unscrew the collection tube and use spoon attached to lid to scoop one spoonful of stool (only 1g or 1mL is needed)	6. Unscrew the collection tube and use spoon attached to lid to scoop one spoonful of stool (only 1g or 1mL is needed)

Be sure no toilet paper is mixed in with your stool samples

Prepare Collection Tube

Stool Sample Collection #1	Stool Sample Collection #2
5. Place spoon into solution of collection tube and screw on tightly. Mix vigorously by shaking or inverting	7. Place spoon into collection tube and seal completely

Reminder: Do NOT store stool samples in the freezer

3 Part Three: Blood Sample Collection

Preparation

1. Wash hands thoroughly with soap and warm water
2. Device will be placed on upper arm - remove excess hair if needed
3. Collect all needed items and remove device from tray
4. Twist off cap from compatible tube; press tube into device until snug with fill lines facing out
5. Follow heat pack instructions to activate; rub heat pack or hand up and down shoulder for 2 mins to warm and increase blood flow
6. Clean area with alcohol pad and allow to dry

2min

Collect Sample

7. Remove clear cover from red button; peel tab away from device and stick device to upper arm (hang arm straight down at side)
8. Press button all the way down ONCE and release
9. Set timer for 5 minutes (Note: blood may not appear for first 1-2 minutes; remove device after 5 minutes or sooner if blood reaches the top line before 5 minutes)

5min

Note: blood sample collection instructions and link to demo video are also available within Tasso+ kits

Part 3 continued & shipping instructions on back

THE ARIZONA
CoVHORT GI
SAMPLE COLLECTION KIT
INSTRUCTIONS

3
Continued

Removal & Packaging

10. Remove device by peeling from one side
11. Remove tube from device with a slight twist and pull down
12. Snap cap carefully onto tube; invert tube per manufacture labeling (10x)
13. Repeat steps 2-12 of Part 3 with second Tasso+ device on other arm
14. Wash hands after use

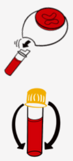

Reminder: Do NOT store blood samples in the freezer

4
Part Four:
Shipping Preparation

Sample Packaging

1. <u>Blood samples</u>: place 1st collection tube into specimen bag from Tasso+ kit (orange bag) and seal completely. Do **not** remove absorbent sheet
2. Repeat step 1 for the 2nd tube using the specimen bag from the 2nd Tasso+ kit
3. <u>Stool samples</u>: Place both stool collection tubes (2) into the specimen collection bag (red bag) and seal completely
4. Complete biospecimen survey

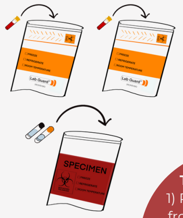

Mailer Packaging

1. Remove all remaining packaging (if any) from one of the Tasso+ boxes. Remove frozen ice pack from freezer ⚠ and place in empty Tasso+ box
2. Place all specimen bags (3) into Tasso+ box on top of ice pack. Close and seal box. The other Tasso+ box can be discarded
3. Place Tasso+ box into bubble mailer and seal completely
4. Place bubble mailer and completed biospecimen survey into FedEx pak and seal completely

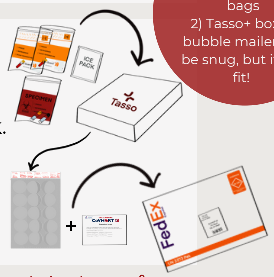

TIPS & NOTES:
1) Remove extra air from biospecimen bags
2) Tasso+ box in bubble mailer will be snug, but it will fit!

Reminder: please ship within 24 hours of blood sample collection (early in the week if possible) & do not remove ice pack from freezer until you are ready to drop package off at FedEx

5
Part Five:
Shipping

Find Drop-Off Location

Go to the link below or scan the QR code to find an accepted FedEx drop-off location to ship your package free of charge:
https://local.fedex.com/en-us/az/

Return Sample to FedEx

Drop off package at FedEx location of choice

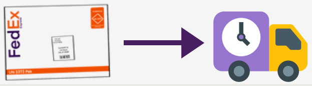

?
What's Next?

Next Steps

Once we have received your completed collection kit, you will receive your $50 electronic gift card via email

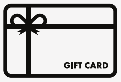

Questions ?

Call us at 520-626-1678 to leave a message or email us at CoVHORT-GI@arizona.edu

Thank you for participating in our study!

Your participation is invaluable in helping us learn more about potential long-term health effects of COVID

ARIZONA
