# Supplementary material for: Determining the incidence, risk factors and biological drivers of irritable bowel syndrome (IBS) as part of the constellation of postacute sequelae of SARS-CoV-2 infection (PASC) outcomes in the Arizona CoVHORT-GI: a longitudinal cohort study
Source: BMJ Open. 2025 Jan 30;15(1):e095093. doi: 10.1136/bmjopen-2024-095093 (PMC11784208; doi:10.1136/bmjopen-2024-095093)
Supplement: online supplemental file 3 [file bmjopen-15-1-s003.pdf]

THE ARIZONA

CoVHORT GI

SAMPLE COLLECTION KIT

CONTENTS

Content Checklist

Ensure your kit contains the following items:

- (1) Fecal collection tube
- (1) DNA/RNA Shield fecal collection tube
- (2) Tasso+ blood sample collection kits
- (1) Pair of disposable gloves
- (1) Commode (toilet hat)
- (1) Ice pack
- (1) Specimen sealable bag
- (1) Insulated bubble mailer
- (1) FedEx pak (poly mailer)
- (1) Biospecimen survey

If any of these items are missing from your kit, please call us at (520) 626-1678 or email us at CoVHORT-GI@arizona.edu

Fecal collection tube (1)

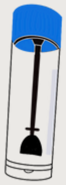

DNA/RNA Shield fecal collection tube (1)

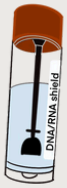

Tasso+ blood sample collection kit (2)

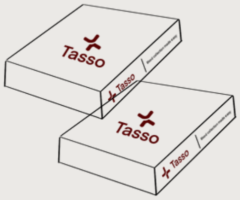

Disposable gloves (1 pair)

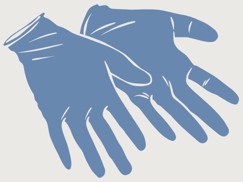

Commode (toilet hat) (1)

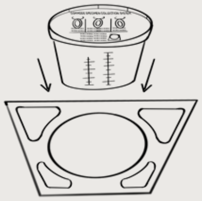

Ice pack (1)

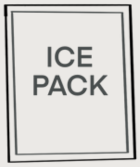

Specimen sealable bag (1)

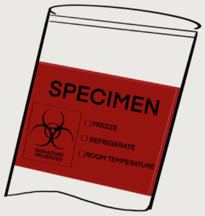

Insulated bubble mailer (1)

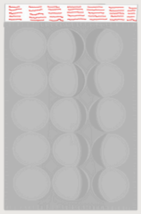

FedEx pak (poly mailer) (1)

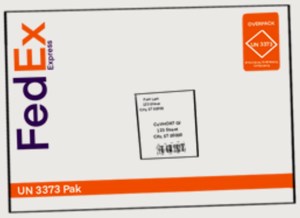

Biospecimen survey (1)

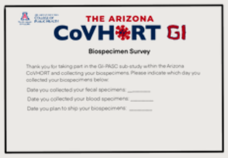

ARIZONA

THE ARIZONA

CoVHORT GI

SAMPLE COLLECTION KIT

CONTENTS

Tasso Kit Contents

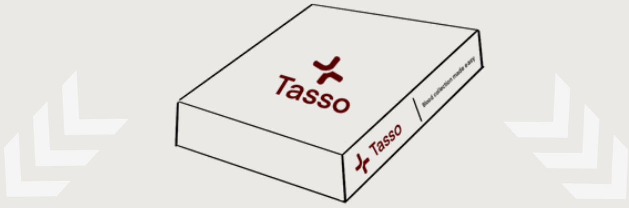

Tasso device

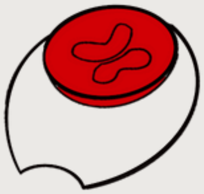

Test tube

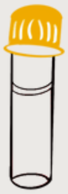

Gel warmer

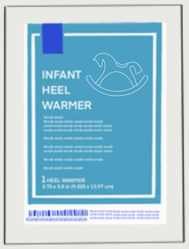

Alcohol pad

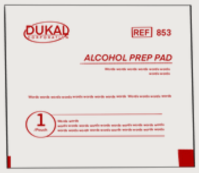

Bandaid

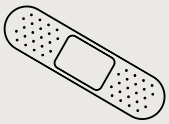

Specimen sealable bag

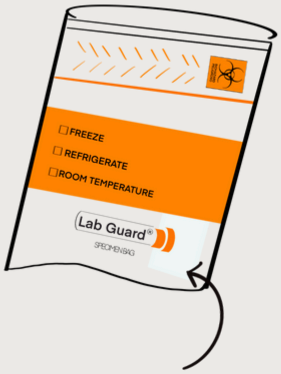

Absorbent sheet - do not remove

Thank you for participating in our study!

Your participation is invaluable in helping us learn more about potential long-term health effects of COVID

ARIZONA
