# Supplementary material for: Determining the incidence, risk factors and biological drivers of irritable bowel syndrome (IBS) as part of the constellation of postacute sequelae of SARS-CoV-2 infection (PASC) outcomes in the Arizona CoVHORT-GI: a longitudinal cohort study
Source: BMJ Open. 2025 Jan 30;15(1):e095093. doi: 10.1136/bmjopen-2024-095093 (PMC11784208; doi:10.1136/bmjopen-2024-095093)
Supplement: Supplementary file 4 [file bmjopen-2024-095093.reviewer_comments.pdf]

PEER REVIEW HISTORY

BMJ Open publishes all reviews undertaken for accepted manuscripts. Reviewers are asked to complete a checklist review form and are provided with free text boxes to elaborate on their assessment. These free text comments are reproduced below.

ARTICLE DETAILS

Title (Provisional)

Determining the incidence, risk factors and biological drivers of Irritable Bowel Syndrome (IBS) as part of the constellation of Post-Acute Sequelae of SARS-CoV-2 infection (PASC) outcomes in the Arizona CoVHORT-GI, a longitudinal cohort study

Authors

Pogreba Brown, Kristen; Austhof, Erika; McFadden, Caitlyn M; Scranton, Caroline; Sun, Xiaoxiao; Vujkovic-Cviji, Ivan; Rodriguez, Dominic; Falk, Laura; Heslin, Kelly M.; Arani, Naga Sai Gayatri Naga Sai Gayatri Arani; Obergh, Victoria; Bessey, Kate; Cooper, Kerry

---

This article was not externally reviewed at BMJ Open. Protocols that have been independently assessed prior to submission to BMJ Open are usually fast-tracked to publication on the grounds that further substantial changes will not be possible. This independent assessment will usually be external, independent review for both a substantial grant award from a non-commercial or government funder and ethics approval.
